# The Lebanese Society for Infectious Dieseases and Clinical Microbiology

**DOI:** 10.1186/1753-6561-5-S6-P3

**Published:** 2011-06-29

**Authors:** JE Mokhbat, The Lebanese Society for Infectious Diseases and Clinical Microbiology

**Affiliations:** 1 Medicine, Lebanese American University, Beirut, Lebanon

## Introduction / objectives

The purpose is to introduce the Lebanese Society for Infectious Diseases and Clinical Microbiology (LSIDCM).

## Methods

The Society was established in 1992.

## Results

It includes 46 infectious diseases physicians as well as clinical microbiologists. The members are spread across the country. All physicians are involved in managing infection control programs in their respective hospitals. So far the Society has organized twelve national and regional congresses on infectious diseases and clinical microbiology. Over the last 4 years, a yearly course on infection control is offered during each congress and many national and international speakers are involved.

## Conclusion

The LSIDCM is currently working on developing national guidelines for the management and control of infectious diseases, national antibiotic usage monitoring, monitoring of antibiotic resistance on a national background and the establishment of a national registry of health care associated infections.

## Disclosure of interest

None declared.

